# Decoding pathology: the role of computational pathology in research and diagnostics

**DOI:** 10.1007/s00424-024-03002-2

**Published:** 2024-08-03

**Authors:** David L. Hölscher, Roman D. Bülow

**Affiliations:** 1 Department for Nephrology and Clinical Immunology, RWTH Aachen University Hospital, Pauwelsstraße 30, 52074 Aachen, Germany; 2 Institute for Pathology, RWTH Aachen University Hospital, Pauwelsstraße 30, 52074 Aachen, Germany

**Keywords:** Deep learning, Pathomics, Digital pathology, Segmentation, Regression, Classification

## Abstract

Traditional histopathology, characterized by manual quantifications and assessments, faces challenges such as low-throughput and inter-observer variability that hinder the introduction of precision medicine in pathology diagnostics and research. The advent of digital pathology allowed the introduction of computational pathology, a discipline that leverages computational methods, especially based on deep learning (DL) techniques, to analyze histopathology specimens. A growing body of research shows impressive performances of DL-based models in pathology for a multitude of tasks, such as mutation prediction, large-scale pathomics analyses, or prognosis prediction. New approaches integrate multimodal data sources and increasingly rely on multi-purpose foundation models. This review provides an introductory overview of advancements in computational pathology and discusses their implications for the future of histopathology in research and diagnostics.

## Introduction

The way histopathology analyses are carried out remained similar for close to two centuries. This classic way typically involves manually assessing specific patterns of injury (e.g., diffuse infiltration of neutrophilic granulocytes) or counting histological objects (e.g., mitoses). However, these manual quantifications and assessments can be cumbersome, low-throughput, error-prone, and subject to inter-observer variability—a hindrance to developing both diagnostics and research further.

The introduction of digital pathology is a transformative development for overcoming these challenges and developing pathology further towards precision medicine. Digital pathology has enabled the introduction of computational methods to pathology, a discipline termed computational pathology [1]. Broadly speaking, computational pathology encompasses computational methods used to analyze patient specimens for diseases. A main branch and topic of this review is computational histopathology that focuses on computational methods applied to digital histopathology images.

Benefits in computational histopathology mainly arise from using artificial intelligence (AI), especially deep learning (DL)–based techniques. DL is a subspecialty of machine learning (ML, a subspecialty of AI) that makes use of artificial neural networks (ANNs) [63]. ANNs, in short, are multilayer functions that progressively transform input data to produce a desired output. During training, the internal parameters of an ANN are automatically updated in an iterative process to produce outputs that are increasingly similar to the ground truth (i.e., the desired output) [63]. This is typically done using a training algorithm that takes actions to minimize a loss function. This paradigm allows training ANNs to map a multitude of input–output relationships, meaning ANNs can be trained to perform a task without being explicitly programmed for it. While that is exciting, it is important to keep in mind that DL model predictions are not of a causal nature, but are correlations based on the relations between input and output that were established in the DL model during training.

Typical applications of ANNs in pathology include classification (assigning a categorical label to data, e.g., hepatocellular carcinoma to a histopathology image) [23], segmentation (pixel-level classification; partitioning an image into discrete groups of pixels, typically corresponding to objects, e.g., glomeruli and tubules) [45], and regression (assigning a continuous value to an image, e.g., gene expression) [28].

Significant progress has been achieved using DL-based solutions in histopathology, e.g., inferring molecular alterations with high precision directly from whole slide images (WSI) [55], predicting the origin of cancers of unknown primary [68], predicting therapy response in colorectal cancer [33], or identifying morphometric biomarkers [3, 45].

In this review, we highlight a selection of studies applying deep learning to digital histopathology to achieve a variety of goals. We focus on major applications, i.e., histomorphometry, classification, and regression (Table 1 provides a selection of major studies on these applications in computational pathology).

**Table 1:** Selection of digital pathology studies stratified by organ, task, and application setting, e.g., cancer, non-cancer, or transplant. Studies are sorted by year of publication

Organ	Task	Setting	Study
Kidney	Classification	Cancer	Ghaffari et al. (2023) [38], Ghaffari et al. (2022) [61], Lu et al. (2021) [69]
	Regression		Shao et al. (2021) [94], Wulczyn et al. (2020) [115]
	Histomorphometry	Non-cancer	Hölscher et al. (2023) [45], Lucarelli et al. (2023) [67], Klinkhammer et al. (2022) [59], Bouteldja et al. (2021) [13], Jayapandian et al. (2021) [50], Ginley et al. (2021) [39]
		Transplant	Yi et al. (2023) [117]
	Classification	Transplant	Kers et al. (2022) [58]
Heart and vasculature	Histomorphometry	Non-cancer	Droste et al. (2023) [25], Peirlinck et al. (2019) [80], Fayyaz et al. (2018) [31], Fry et al. (2014) [35]
	Classification	Transplant	Seraphin et al. (2023) [93]
Liver and intestine	Classification	Cancer	Wagner et al. (2023) [108], Försch et al. (2023) [33], Chen et al. (2020) [18], Liao et al. (2020) [65], Kather et al. (2019) [57]
	Regression		Shao et al. (2021) [94], Wulczyn et al. (2020) [115], Saillard et al. (2020) [88], Kather et al. (2019) [56], Skrede et al. (2020) [98]
		Non-cancer	Bosch et al. (2021) [12]
	Histomorphometry	Non-cancer	Taylor-Weiner et al. (2021) [105], Suppli et al. (2019) [104]
		Transplant	Sun et al. (2020) [103]
Connective tissue	Histomorphometry	Non-cancer	Recker et al. (2020) [85], Reyes-Fernandez et al. (2019) [86], Evenepoel et al. (2017) [30], Behets et al. (2015) [8], Smith et al. (2014) [99], Osman et al. (2013) [77]
Brain	Regression	Non-cancer	Marx et al. (2023) [71]
	Histomorphometry	Non-cancer	Boeckh-Behrens et al. (2016) [10], Boeckh-Behrens et al. (2016) [9]
Lung	Classification	Cancer	Kanavati et al. (2020) [54], Gertych et al. (2019) [37], Coudray et al. (2018) [23]
	Histomorphometry		Wang et al. (2017) [109]
	Regression		Shao et al. (2021) [94], Wulczyn et al. (2020) [115], Yao et al. (2020) [116]
Reproductive system	Histomorphometry	Cancer	Whitney et al. (2018) [113], Leo et al. (2016) [64]
	Regression		Shi et al. (2023) [95], Wulczyn et al. (2020) [115]
Breast	Histomorphometry	Cancer	Amgad et al. (2023) [3]
	Classification		Wang et al. (2022) [111], Wang et al. (2021) [110], Qu et al. (2021) [83], Ektefaie et al. (2021) [27], Arujo et al. (2017)
Prostate	Classification	Cancer	Raciti et al. (2023) [84], Bulten et al. (2022) [15], Pantanowitz et al. (2020) [78], Bulten et al. (2020) [16]

## Histomorphometry

**Fig. 1:**
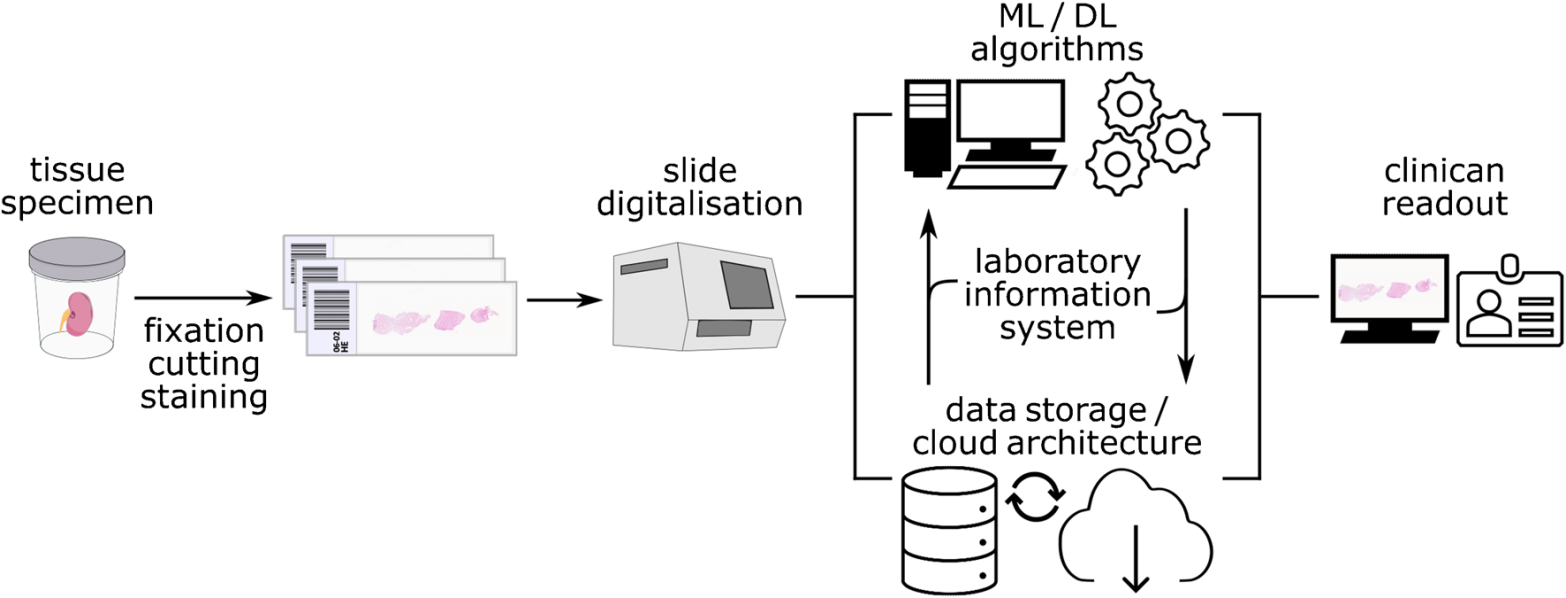
Digital pathology ecosystem encompassed in standard tissue analysis workflows. Regular tissue processing is followed by digitalization of tissue slides into whole slide images (WSIs). WSIs are the main data resource for digital pathology ecosystems in which they are stored, associated with other input data in the laboratory information system (LIS), and analyzed by machine or deep learning algorithms. Eventually, clinicians are provided with a bundle of extensive resources including the digitized tissue slide for informed decision making. ML, machine learning; DL, deep learning

The hypothesis that form follows function has been extensively investigated in medical specialties such as anatomy or physiology. In principle, investigating the shape of a structure of interest can lead to a deeper understanding of its specific function. Tissue analysis based upon the form of tissue compartments or structures to derive novel insights into their functionality has been practiced for over 40 years [21]. However, these measurement and quantification tasks were mostly done manually, e.g., using digital planimeters [34] or drawing tubes mounted on microscopes [70]. Thus, these techniques were limited by their time-consumptive nature, technical capabilities, and inter-observer variability [22]. Computational pathology algorithms have evolved rapidly over the recent years, especially fueled by the digitalization of tissue slides (Fig. 1). Multiple commercial and open source software for image analysis have implemented histomorphometry workflows, e.g., MATLAB [99] or ImageJ—Fiji [25]. Whether these workflows are based on readily available plugins, self-coded macros, or in-built deep learning algorithms, they all perform the task of semantic segmentation to delineate structures of interest.

### Semantic segmentation

**Fig. 2:**
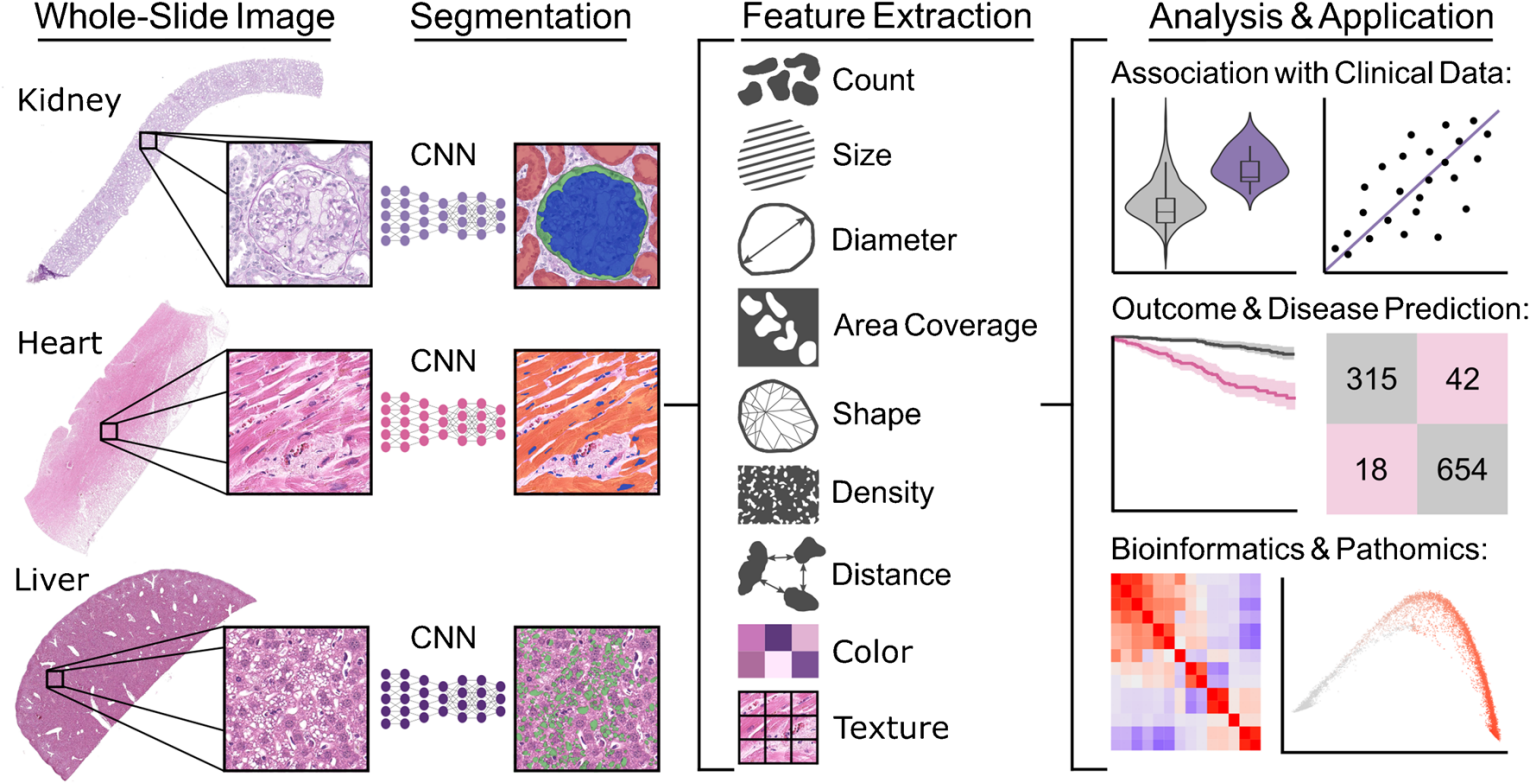
Histomorphometry techniques for different organ systems including analysis applications. Convolutional neural networks (CNNs) are applied to image patches of organ histology for segmentation of regions of interest. These image masks are then used for calculation of morphometric features which can be implemented in downstream analyses. CNN, convolutional neural network

Histopathology segmentation tasks represent the precise delineation of complex tissue structures. Segmentation models produce pixel-level image masks for desired structures and compartments in WSIs. These masks allow for the extraction of hand-crafted features by further algorithms. Features represent different attributes of the structure of interest and can range from simple and explainable distance or area measures to complex readouts of texture (e.g., entropy, contrast, homogeneity) or image moments which range beyond the capabilities of the human eye. Extracted features can then be associated with clinical data such as laboratory values [67], be implemented in disease or outcome prediction models [117], and in downstream bioinformatics analyses [45] (Fig. 2). Feature importance for specific tasks can be computationally investigated, potentially allowing to identify novel associations between form and function.

The quality and meaningfulness of features are inherently dependent on precise segmentation masks. To generate precise segmentation results, models need to learn based on a ground truth, i.e., the human prespecification on how structures of interest should be outlined, which often remains a laborious task. Therefore, an inherent challenge for segmentation in high-throughput settings, where manual control is not feasible, is continuous quality control of the segmentation output. Methods such as reverse classification accuracy [87] or anomaly detection [36] can help to automatically assess segmentation accuracy. As segmentation models require manual oversight as well as handcrafted annotations enhancing the learning process, they have to be adapted to each specific research setting. However, this also allows for considerable flexibility, as segmentation models can be applied to various resolutions and imaging modalities, such as bright-field, fluorescence, or electron microscopy. Depending on the respective task, this enables the segmentation of various levels of tissue architecture such as functional units, compartments, extracellular space, or nuclei.

### Kidney

Kidney microanatomy consists of many diverse and complex structures such as glomeruli, different sections of tubules, the interstitium, and vessels. In recent years, considerable efforts have been made to accurately segment these structures in human and animal specimens for different histological stains, laying the groundwork for histomorphometry analysis [13, 44, 45, 50]. In diabetic nephropathy, one of the most prevalent causes of chronic kidney disease, segmentation models could accurately assess glomerulosclerosis as well as interstitial fibrosis and tubular atrophy (IFTA), which represent important hallmarks of chronic kidney disease [39]. The model’s quantification of IFTA reached high agreement with experienced renal pathologists (intra-class correlation coefficient of 0.94 when including the ANN as an observer) while being more time-efficient compared to human manual annotations.

Furthermore, detailed histomorphometry analysis can reveal alterations of tissue architecture that were not captured by functional and laboratory parameters. Klinkhammer et al. implemented two common murine chronic kidney disease models to analyze kidney disease recovery [59]. Interestingly, while kidney function measured by glomerular filtration rate normalized back to baseline 2 weeks after recovery, histomorphometry analysis of tubular architecture in more than half a million tubules revealed persisting dilation and atrophy of tubules leading to further nephron loss.

### Liver

In liver histology, histomorphometry is mainly implemented for quantification of steatosis, i.e., fat accumulation in liver cells, and fibrosis. Both are important histopathological features of tissue remodeling pathways in steatohepatitis leading towards liver cirrhosis. Accurately quantifying liver steatosis and fibrosis allows for monitoring of disease progression and even therapy response for novel drugs. In a reanalysis of three randomized clinical trials including core-needle biopsy samples from 3662 patients with non-alcoholic steatohepatitis (NASH), Taylor-Weiner et al. developed a novel score summarizing fibrosis patterns on patient level termed the Deep Learning Treatment Assessment (DELTA) liver fibrosis score which accurately captures changes in tissue remodeling after anti-fibrotic therapy [105]. Broad implementation of the DELTA liver fibrosis score could facilitate standardized and reproducible assessment of treatment response in future trials regarding anti-fibrotic agents for liver disease. Liver steatosis quantification can also be implemented in liver transplant workflows, allowing for better organ allocation. Sun et al. developed a deep learning-based segmentation model for quantifying steatosis in frozen liver donor sections which could outperform the estimations of an on-service pathologist thus potentially leading to 9% fewer unnecessarily discarded liver transplants [103].

### Heart and vasculature

Cardiomyocyte morphology is a key factor for understanding the pathophysiology of heart failure. Segmentation of cardiac myocytes in a murine model of transaortic constriction (TAC) as well as in autopsy samples of humans with aortic (valve) stenosis revealed similar increases in cardiomyocyte area and decreases of capillary contacts per area of cardiomyocytes signaling myocyte hypertrophy and microvascular rarefaction in pressure overload-induced heart failure [25]. Histomorphometry of cardiomyocyte hypertrophy on an electron microscopy scale in a guinea pig TAC model could also be linked to changes in intra- and transcellular electric conductance [35]. Fry et al. demonstrate how, although the topological arrangement of cells is retained in left ventricular hypertrophy, an increase in lateral cell-to-cell connections and intercalated disk space leads to altered three-dimensional cardiac action potential conduction velocity. Myocyte hypertrophy can not only lead to altered electrophysiology but also result in hemodynamic changes. In a multi-scale computational model of heart failure including cardiomyocyte histomorphometry, ventricular volume overload in pig hearts was directly associated with lengthening of cardiac myocytes leading to a decrease in measured ejection fraction during echocardiography [80].

Not only cardiac remodeling but also vascular remodeling in pulmonary hypertension is known to contribute to heart failure. Fayyaz et al. investigated vessel histomorphometry for pulmonary arteries, veins, and small indeterminate vessels in human autopsy or surgery specimens [31]. Interestingly, the severity of pulmonary hypertension and presence of heart failure were more determined by increases in intimal thickness of veins and small indeterminate vessels rather than the remodeling of arteries.

### Soft tissue

Similar to the analysis of cardiac myocytes, skeletal muscle fibers can also be analyzed by histomorphometry allowing for characterization of different fiber types and muscle metabolism [99]. Quantifying the fiber size of human deltoid and pectoral muscle enables spatial analysis of location-specific muscle morphometry revealing different age-, sex-, and myopathy-related patterns of atrophy and hypertrophy [86].

In fatty tissue, segmentation of adipocytes has been widely established due to their rather simple shape and configuration. Osman et al. developed a MATLAB algorithm for high-throughput batch quantification of white fat morphology in high-fat diet-fed mice [77]. They precisely demonstrate how adipocytes significantly increase in size in high-fat diet-fed mice, although this increase is overestimated when using manual annotation methods. The authors propose that this is due to changes in adipocyte shape which cannot be accurately assessed by manual annotation methods.

### Blood clots

In recent years, interest has sparked in analysis of cerebral blood clots retrieved during mechanical thrombectomy which can provide diagnostic insights into clot etiology as well as patient outcome [10]. This field can be enhanced by automated histomorphometric composition analysis. Automated quantitative analysis of the relative fractions of clot components (red or white blood cells, platelets, and fibrin) could differentiate between clot compositions of cardioembolic and noncardioembolic etiology in a retrospective study of 145 stroke patients. Further analysis of cryptogenic stroke thrombi revealed similar composition characteristics to cardioembolic thrombi, but distinct from noncardioembolic thrombi, supporting the hypothesis that many cryptogenic strokes are in fact cardioembolic [9].

### Subcellular structures

Next to analysis of compartments or structures, histomorphometry can also expand to the level of subcellular organelles, mainly the segmentation and quantification of nuclei. Histomorphometry of nuclei is especially interesting for cancerous tissue. Nuclear features such as chromatin clumping have been identified as predictive of patient risk, overall survival, and recurrence in various cancer types such as breast [3, 113] or lung cancer [109].

Also considerable efforts have been made to enable segmentation of cellular organelles in electron microscopy. Heinrich et al. were able to segment over 30 different classes of cellular organelles, e.g., endoplasmic reticulum, microtubules, or ribosomes on nanometer level in reconstructed three-dimensional electron microscopy images [43]. This opens up a whole new resolution of histomorphometry analysis providing insights into spatial interactions and organelle architecture.

### Pathomics

Due to the wide range of different features to characterize segmented tissue, feature extraction can generate large data frames containing thousands of segmented structures and all extracted features for each respective structure. These large data frames resemble datasets generated by molecular omics workflows, such as single cell transcriptomics and can be used for dimensionality reduction or trajectory inference analysis (Fig. 2). Hence, the term of pathomics has been proposed to characterize the data mining of histopathology images [14, 32, 42, 45]. Pathomics enables precise characterization of whole tissue architectures, but can also be applied to cancer specimens. Mining of pathomics data from cancer cells and their microenvironment allows for precise quantification of stromal, nuclear, or immune features thus enabling new prognostic biomarkers for breast [3] or colon cancer [32]. Pathomics is inherently spatial due to the traceable location of all segmented structures and could provide a missing link on how molecular processes influence tissue architecture and organ function [14].

Downstream analysis of datasets can be augmented by various readily available machine learning techniques. By linking pathomics with clinical and histopathological context, combined datasets can be applied to multivariate analyses for discovery of novel associations or developing for clinical practice. Established machine learning applications include support vector machines which find an optimal hyperplane that best captures the maximum differences of provided groups of interest. Depending on the dimensionality of the datasets, the hyperplane resembles a line (two-dimensional), a plane (three-dimensional), or more abstract geometric shapes (n-dimensional). Furthermore, tree-based models such as random forests, boosted trees, and classification and regression trees as well as more traditional statistical models such as linear, logistic, ridge, and lasso regression can be applied to pathomics data. For discovery of novel associations, k-means or hierarchical clustering algorithms can group statistically similar data points together and are already widely known in other omics fields. Pathomics has already been combined with other established omics such as genomics [3] or transcriptomics [104] in large-scale multi-omics studies and multiple applications and platforms exist for analyzing pathomics data [46, 82]. In the future, further bioinformatics methods have to be implemented to link multi-omics datasets to relevant patient-level outcomes [52].

## Classification

**Fig. 3:**
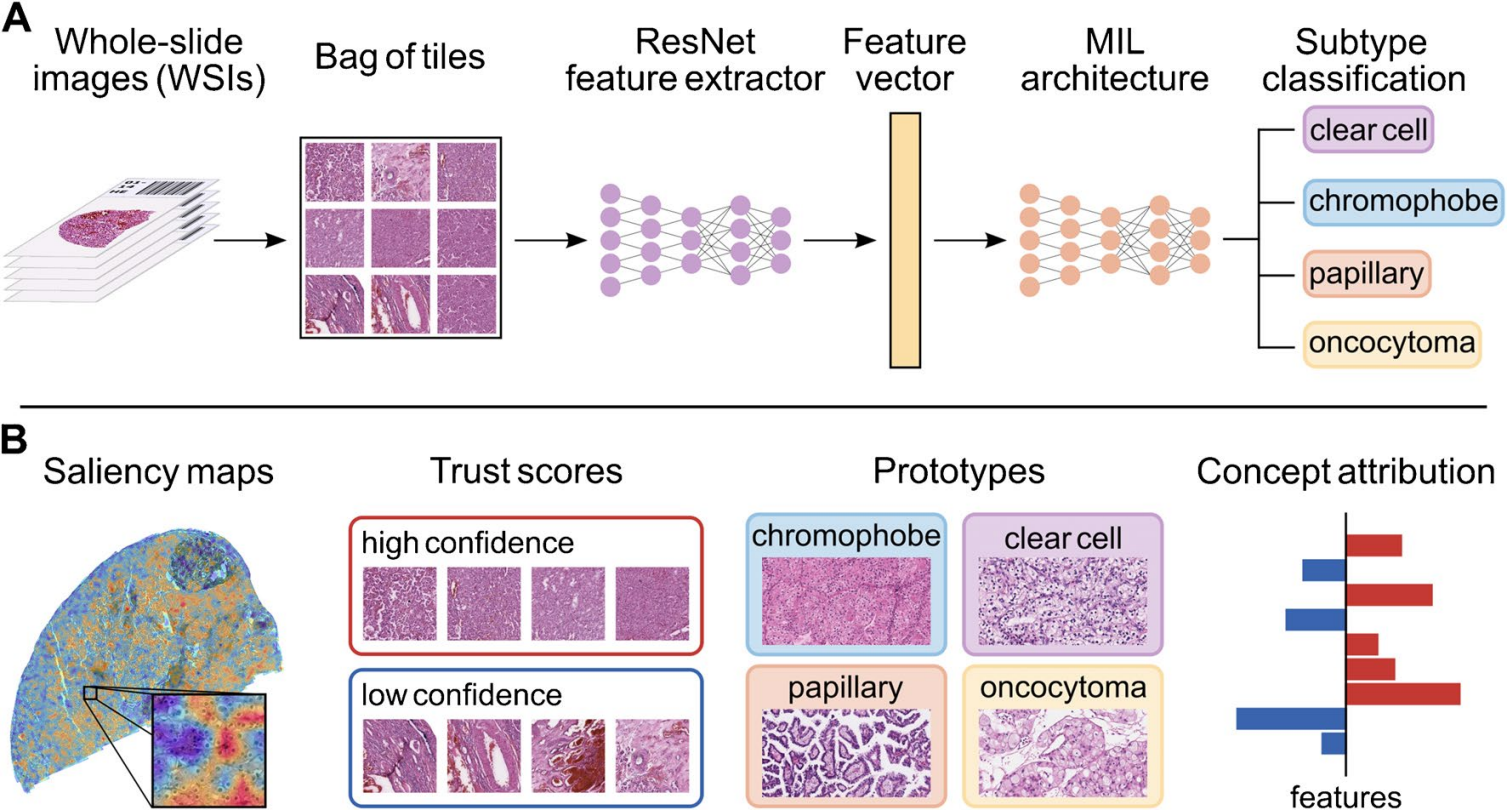
Deep learning–based classification of histopathology and approaches to interpreting the basis for classification. **A** Whole slide images (WSIs) are tessellated into image tiles. In a multiple instance setting, typically, a pre-trained deep learning model is used as a feature extractor that transforms each image tile into a high level feature vector which is used by another model to formulate a prediction. **B** These predictions are usually opaque, but techniques exist to make them interpretable (explainable artificial intelligence (XAI)). Among the most popular XAI techniques in pathology are saliency maps, trust scores, prototype examples, and concept attribution. We thank Yu-Chia Lan, M.Sc., for providing a saliency map visualization for this figure

Formulating a diagnosis in digital pathology can be framed as a classification task, i.e., assigning one or several categorical label(s) to WSIs. Consequently, many studies in computational histopathology have focused on end-to-end classification tasks [55, 68, 96]. In most studies, the ground truth used for training ANNs consists of WSI-label pairs, meaning that one or several WSI are associated with a categorical label, e.g., “Clear Cell Renal Cell Carcinoma” (Fig. 3). However, due to hardware constraints and data size, WSIs are typically tessellated into smaller image tiles. Because of that, not every image tile contains histological features that are diagnostic for the case label, e.g., when only a small part of the slide contains cancerous tissue.

An often used approach to tackle this challenge is called multiple instance learning (MIL). In short, not every image tile in MIL inherits the case label, but the entire collection of tiles making up a WSI gets assigned a label. In a binary classification setup, this means that the entire collection of tiles is negative, if all tiles in the collection are negative, and positive, if at least one tile in the collection is positive [17]. This allows for efficient processing of WSIs in weakly supervised settings (e.g., one label per slide), which is beneficial for dataset generation, since weak labels are inherently easier to retrieve than strong labels. MIL and other classification techniques have been investigated for a multitude of pathology classification tasks in several organs. Although there are some exceptions [58, 93], most classification studies in computational pathology are concerned with cancer specimens. Due to the sheer number of studies published in the field, we had to limit ourselves to a selection of studies applying DL-based tools to major cancer types and pan-cancer approaches.

### Deep learning–based classification of lung *cancer*

With an estimated 2.2 million new cases in 2020, carcinomas of the lung are among the most common types of cancer in both men and women and are the leading cause of cancer deaths [102]. Classification of histopathological images as normal or lung carcinoma (any type) was shown to be feasible using ANNs with AUROCs above 0.973 in a study using 4,704 WSI of lung histopathology [54]. This study investigated both a fully supervised and a weakly supervised approach, showing that the weakly supervised approach consistently showed better performance than the fully supervised approach [54]. Another study investigated prediction of mutations in the following genes: *KRAS*, *TP53*, *EGFR*, *STK11*, *FAT1*, and *SETBP1* with AUROCs higher than 0.733 [23]. *STK11* mutations could be predicted with the highest accuracy (AUROC 0.85). In addition, the authors investigated ANNs for distinguishing between lung squamous cell carcinomas and adenocarcinomas, a task for which they achieved high accuracy (AUROC of 0.97) [23]. Lung adenocarcinomas can present with different growth patterns. A lightweight (i.e., less complex) ANN was shown to be trainable to distinguish solid, cribriform, micropapillary, and acinar growth patterns of lung adenocarcinomas [37]. However, the primary use case of such a model would likely be delineating areas with different growth patterns, but the resolution of the classification heatmaps here is not on pixel level, but on tile level, which would lead to an inaccurate delineation, a limitation that could be circumvented using a segmentation model.

### Deep learning–based classification of prostate *cancer*

With an estimated 1.41 million new cases in 2020, prostate cancer is one of the most common types of cancer worldwide and a significant burden to global health [118]. Diagnostic criteria of prostate carcinomas are well defined, and its grading scheme—the Gleason grades—is defined by tissue architecture [48]. The combination of case abundance and well-defined histopathology makes prostate cancer an ideal entity for developing computational pathology tools. Automated Gleason grading was demonstrated in a large study including 5759 biopsies from 1243 patients [16]. The model showed excellent agreement with the ground truth (quadratic Cohen’s Kappa of 0.918), indicating its usefulness for clinical diagnostics [16].

High accuracy of prostate cancer and perineural invasion detection was achieved in another study [78]. Importantly, this was the first study reporting identification of a prostate cancer case by a DL system that was missed by a pathologist [78]. In the large Prostate cANcer graDe Assessment (PANDA) challenge that included approximately 11,000 WSI, many teams achieved accuracies on par or better than pathologists (e.g., sensitivity for tumor detection of 99.7% with specificity of 92.9%), mostly using end-to-end techniques with case level information only [15]. Given these impressive performances, it is not surprising that there are several commercial DL-based tools to be used in prostate cancer histopathology diagnostics that were already shown to help increase sensitivity and specificity of diagnostics [84].

### Deep learning–based classification of breast *cancer*

Breast cancer is the most common invasive cancer in women with approximately 2.3 million new cases in 2020 [5]. Although the primary goal in computational breast pathology has been to detect and outline tumors both in mammary tissue and lymph nodes [26], histopathology classification studies using DL-based tools were performed relatively early as well [4, 112].

Automatic classification of lobular versus ductal invasive carcinoma of the breast with an accuracy of 94% was achieved with a neural network on tissue microarray cores [24]. Similarly, another study achieved an area under the receiver operating characteristics curve (AUROC) of > 95% in independent cohorts for breast cancer subtype classification using ANNs on WSI [27].

DeepGrade, an ensemble of multiple neural networks, could be used to re-stratify breast cancer cases classified as G2 (Nottingham grading system), corresponding to intermediate differentiation [111]. The DeepGrade-based stratification (Grade 2 high/low) was an independent predictor of patient survival, indicating usefulness of such stratification [111].

Point mutations in *RB1*, *CDH1*, *NOTCH2*, and *TP53* (AUROCs > 0.729) could be directly inferred from histology using a classification DL network [83]. Another study showed that using an ANN, a germline *BRCA1/2* mutation could be predicted directly from digital histopathology [110]. Although these molecular predictors are of interest, the accuracy is not high enough to use them instead of molecular investigations.

### Deep learning–based classification of colorectal *cancer*

Predicting microsatellite instability (MSI) from WSI of colorectal cancer specimens using DL was one of the early landmark studies in computational pathology [57]. In the meantime, this was reproduced multiple times using a variety of approaches. DL-based MSI prediction tools have reached clinical grade performance (sensitivity of 0.99 and negative predictive value of 0.99), allowing the use of such models as screening tools before performing molecular analyses [108].

In addition to deficiencies in mismatch repair genes, other mutations were also shown to be predictable from WSIs of colorectal cancer specimens, including *APC*, *KRAS*, *PIK3CA*, *SMAD4*, and *TP53* [49]. However, the predictive performance in this study was not high enough (AUROC 0.693–0.809) to justify using the model in clinical or research practice for this specific task.

The consensus molecular subtype (CMS) of colorectal cancer is a gene expression–based subtyping system with biological interpretability and prognostic implications [41]. The CMS was predicted directly from WSIs with good accuracy (AUROC 0.84) using an ANN [97]. Cox-proportional hazards analyses revealed similar predictive performances for the molecular and image-based CMS classification [97]. Thus, such an image-based classification could help cut costs for molecular analyses while at the same time providing similar predictive performances.

A multimodal DL-based approach using different immunostains (CD4, CD8, CD20, CD68) was used to predict the relapse free survival status after 3 years in a multicenter cohort of colorectal cancer patients (*n* > 1000) with good accuracy [33]. Data from these stains were integrated into a score termed AImmunscore by the authors, which proved to be a strong and independent predictor of prognosis in colorectal cancer patients [33].

### Multi-*cancer* classification studies

In addition to studies focusing on DL applications for one type of cancer, several studies investigated DL approaches for multiple cancer types. Cancers of unknown primary (CUP), i.e., manifestations of cancerous tissue the origin of which is not known, are a major diagnostic challenge. In a landmark study, Lu et al. developed a DL model to simultaneously predict whether a tumor is primary or metastatic and predict its site of origin [68]. The model reached a top-1 accuracy of 0.83 and a top-3 accuracy (meaning the correct class is in the three predictions with the highest probability score) of 0.96, which is excellent [68]. Such a model could potentially have a major assistive impact for pathologists. An in-depth analysis of the usefulness of predictions in a prospective setting when pathologists cannot guess the site of origin from morphology would be highly interesting.

In an elegant study, an ImageNet-pretrained Xception model that was fine-tuned on colorectal cancer histopathology was used to extract features from WSI tiles of lung adenocarcinomas, head and neck squamous cell carcinomas, and colorectal carcinomas [20]. The tiles were then classified into four groups of clusters using unsupervised clustering (K-means) on a subset and nearest neighbor analysis on the remaining tiles. Tiles from different clusters correspond to different tissue areas and, interestingly, had different predictive values for mutation prediction, allowing the authors to guess which tissue parts are most informative about mutations [20]. This allows for the development of a more interpretable approach to mutation prediction than classic end-to-end approaches.

## Approaches to interpretability of end-to-end classification models in computational pathology

Predictions made by a DL-based system are typically opaque, i.e., the basis for the prediction is unclear to the user. The field of explainable AI (XAI) aims to develop methods that help interpret and consequently understand the basis of model predictions (for an in-depth review on XAI in pathology, see [81]). Major XAI approaches include saliency maps, trust scores, prototypes, and concept attribution (Fig. 3).

Saliency maps are typically displayed as overlays indicating an estimation of the relevance of an area in the image to the model prediction. A major computational approach for producing saliency maps are gradient weighted class activation maps (Grad-CAM) [91]. Using these heatmap-like visualizations, a user can evaluate whether highlighted areas include morphologically typical variations for the predicted class (Fig. 3). However, when a task is performed for which no typical morphological variations are described (e.g., predicting certain mutations), evaluation of saliency maps can be challenging. Indeed, evaluation of saliency maps is prone to several sources of bias, for example, positive confirmation bias of pathologist expectation [29].

Trust scores help evaluate how confident a model prediction is. Given high model accuracy, this can be helpful for clinical implementation, e.g., to design a strategy for triaging cases which potentially do not require review by a pathologist, or cases that require review or to assist physicians in weighing the model prediction in their decision-making. However, confidence scores can be high for wrong predictions, which limits their validity. This is impressively demonstrated using adversarial attacks [38], where sometimes subvisual changes introduced to an image completely change the predicted class.

Prototypes are representations of instances (e.g., an image tile) that are “archetypal,” i.e., highly representative for the model classes. Often, the most predictive tiles from the patients with the highest prediction scores are depicted (e.g., in [58, 74]). However, synthetic generation of prototypical instances potentially allows more flexible interpretation of prototypes [66].

Concept attribution methods aim to estimate the relevance of manually defined features or concepts for model prediction [72]. These can range from local instance features, such as the circularity of a nucleus to more broad and potentially less understandable features, such as the image texture. It is necessary to select these concepts up front, which is a significant source of bias. However, novel unsupervised methods based on latent space deconvolution might provide a less biased approach to finding relevant concepts [40], although to our knowledge and at the time of writing this review, this has yet to be evaluated in histopathology.

These XAI techniques are important components of computational pathology techniques. They help evaluate bases for predictions and might be used to uncover new statistical correlations between images and a desired output. Importantly, they can help uncover spurious correlations or batch effects, e.g., when an ANN focuses on the background, not the tissue, while delivering good results. As such, they are helpful tools to build trust in model predictions.

## Deep learning regression in pathology

### Time-to-event regression

Regression techniques in digital pathology often encompass time-to-event regression, i.e., analyzing the length of time until the occurrence of a predefined endpoint. The most prominent applications are in cancer histopathology, especially predicting overall survival [56, 94, 98, 115, 116]. The implemented models are often based on end-to-end image regression and can predict the desired endpoint directly from the image input, thus not being dependent on the extraction of hand-crafted morphometric features. However, similar approaches include classification of patients into risk groups which are then implemented as a covariate in a Cox regression model [95]. Other than predicting overall survival, there have been multiple studies on predicting cancer recurrence in breast cancer [95] or hepatocellular carcinoma [88].

### Linear and logistic regression

Nevertheless, regression is not limited to time-to-event analysis, but also includes prediction of quantitative parameters. This can be especially interesting when deep learning models are able to predict quantitative clinical characteristics or pathophysiological alterations from histology. Recent studies have hypothesized that this approach holds advantages over classification workflows due to the continuous nature of measurements. Classification approaches often lead to dichotomization of the desired output value which results in a significant loss of predictive performance. Kather et al. demonstrate that a regression model for predicting homologous recombination deficiency, i.e., a biomarker for genome instability, outperformed traditional classification approaches in five of seven cancer types with similar predictive performance for the other two subtypes [28].

Similar to approaches in cancer pathology, multiple instance learning can be leveraged to accurately predict continuous brain age from post-mortem hippocampal sections [71]. The predicted brain age (HistoAge) unveiled novel determinants of age-related functional decline such as increased model attention to the C2 hippocampal subfield. HistoAge also showed significant associations with clinical features of cognitive impairment that were not found based on epigenetic methylation analysis.

Additionally, liver histology analysis could accurately predict the hepatic venous pressure gradient, an important physiological parameter for the estimation of portal hypertension in liver disease [12]. The proposed analysis method of liver biopsies from patients with compensated liver cirrhosis could quantify important hemodynamic changes which otherwise can only be determined by invasive interventional radiography.

## Routine implementation in research and diagnostics

To implement computational pathology approaches in routine biomedical research and clinical pathology, several aspects must be considered. The most important aspects for implementation in both research and diagnostics likely are accuracy and reproducibility of model predictions [60] which resemble the common quality criteria for biomedical tests and research. However, ease of use is similarly important for distribution of deep learning applications in biomedical research, since many biomedical researchers lack the specific bioinformatics expertise needed to deploy trained models without dedicated available software.

Open source software that enables researchers to train and deploy deep learning models on their own with minimal or no coding expertise required are thus very valuable. Examples of such softwares include QuPath [6], which, e.g., has a StarDist [89] and a Cellpose [101] extension that can be used to perform DL-based cell segmentation. Another prominent example is napari [2], which has over 100 extensions [47] and can be used to access ImageJ features [92], which is highly interesting due to the broad use of ImageJ [90] in the biomedical community. A few other examples include CellProfiler [62], ilastik [100], or Orbit Image Analysis [76]. Given such tools, researchers can design their own deep learning–based biomedical image analysis pipelines without the need to code, potentially even using open source software assisting with downstream analysis of the generated data, such as CellProfiler Analyst [51] or Trigon [46].

To enable comparability of study results, standardization is needed. This is however largely lacking for studies performing pathomics analyses [14]. For the analysis of bone density and structure, histomorphometry is already a well-established application. Here, validated protocols and recognized definitions of structures have already been published [30]. Especially for renal osteodystrophy and osteoporosis, bone histomorphometry has been widely recognized as a valuable method for evaluating skeletal remodeling in clinical trials [8, 85]. Further efforts have to be made for reaching a consensus analysis workflow for other tissues as well to enable similar successes.

**Fig. 4:**
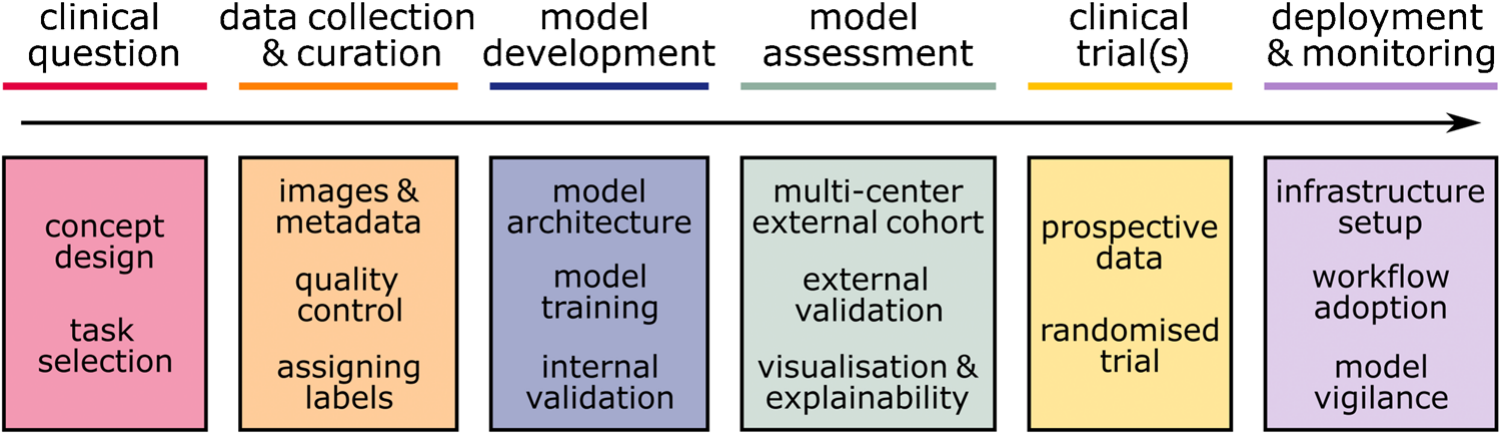
Workflow for implementation of computational pathology algorithms into clinical routine practice. Accurately formulating a clinical question, generating meaningful input data, and selecting an appropriate model architecture for the desired task are crucial for then developing a precise and robust algorithm. Computational pathology algorithms should ideally be validated in external independent cohorts and further evaluated in randomized controlled trials to demonstrate their impact on patient outcomes. After successful implementation in clinical workflows, algorithms need to be continuously monitored and adapted to the collected real-world data which provides important long-term longitudinal information to further improve model performance

Before clinical implementation, a number of development and validation stages should ideally be completed (Fig. 4). Currently, the major hurdles for clinical diagnostic use and implementation are limited digitalization of pathology institutes and the lack of prospective evidence, although some studies emerge that investigate deep learning models in pathology prospectively [7]. Prospective evidence is needed to robustly prove a benefit of using deep learning models both for pathology diagnostics and for clinicians receiving new information (e.g., prognostic information generated by a model). That being said, another significant hurdle is the lack of reimbursement for using deep learning models in diagnostic pathology in many countries, which means, costs associated with buying software must be amortized through gaining efficiency. One reason for the lack of reimbursement might be that designing a reimbursement strategy for using image analysis tools in clinical medicine is challenging. Still, reimbursement concepts are increasingly emerging [79].

## Foundation models in computational pathology

Medical deep learning and also computational pathology show a trend towards using foundation models, i.e., models that are pre-trained on a wide range of data that can be adapted to perform many different downstream tasks (for an in-depth report on foundation models, see [11]). Probably the most well-known example of a foundation model is ChatGPT, based on a generative pre-trained transformer model [75]. In medicine, foundation models have been used primarily for non-imaging data [114], with a prominent example being the AlphaFold model [53].

In computational pathology, foundation models are only starting to appear. This is likely due to the fact that collecting a large and diverse enough dataset of WSI still poses a challenge. Still, some foundation models already exist in computational pathology. Virchow is a model that was trained on 1.5 million WSI of H&E stained tissue sections [107]. This model can be used to develop a cancer detection model with a very high accuracy (AUROC of 0.949 across 17 cancer types). While that is impressive, the Virchow model is not openly available, limiting its useability for the biomedical community. UNI is another foundation model for computational pathology [19]. UNI was shown to surpass the previous state of the art in several computational pathology tasks, e.g., tumor lymphocyte detection, assessed in the ChampKit benchmark [19]. A major advantage of UNI is that it is available for research purposes (for modes of access, see [19]), which will accelerate computational pathology research. CONCH is a visual-language foundation model for computational pathology developed by the same group that developed UNI [19]. CONCH was developed using 1.17 million text-image pairs. The intuition behind additionally using text is that image descriptions contain key information that might be hard to extract for a machine learning model automatically only from the image. The fact that pathologists can extrapolate from a few examples of image-text pairs might be seen as supportive for that intuition. CONCH outperformed several previous approaches across many tasks, such as image classification or segmentation [19]. CONCH is available for research purposes as well.

## Outlook

Computational pathology transformed, transforms, and will further transform the way pathology diagnostics and research is being done. We assume that the current trend towards generalist foundation models will continue. These models will likely foster the development of more accurate specialist models that might even come with a text interface. Importantly, generalist multimodal models could be useful for integrating the vast amount of multimodal medical data produced everyday and perform a number of tasks. An interesting new paradigm is generalist medical AI (GMAI), i.e., multimodal foundation models that can perform many different medical specialist tasks without explicit training [73]. GMAI models would enable interactive procedures, in which physicians, e.g., perform analysis of a WSI image together with a GMAI model by querying the model using text- or voice-based input and receiving text-, voice-, or image-based outputs with explanations. While there currently is no GMAI model available, such a development does not seem far off given the current pace of research in foundation models. However, given the large number of parameters in these models, the computational overhead and consequently energy consumption of these models is potentially enormous and should be considered before large-scale implementation [106].

## Conclusion

The evolution of histopathology from manual, error-prone analyses to the advent of digital pathology and the introduction of computational pathology, particularly computational histopathology, has revolutionized the way pathology specimens are analyzed. The use of AI, specifically deep learning–based techniques, further enhances transformation of pathology, e.g., enabling automated high-throughput classification, segmentation, and regression as well as multimodal data integration with high precision, a significant advance towards precision medicine and decoding the pathologic basis of diseases.

## Data Availability

No datasets were generated or analysed during the current study.
